# Gifts and Gifts

**Published:** 1873-04

**Authors:** Ben. H. Pratt


### THE BISTOURY.

Vol. IX. ELMIRA, N. Y., APRIL, 1873. No. 1.

### Communications.

### GIFTS AND GIFTS.

BEN. H. PRATT, A. M.

It is a pity that Society condemns female labor. Perhaps that's putting it stronger than I mean, for it does not so oppose the literary and vocal and art efforts put forth by the sex. Applause, admiration, honor, respect, wealth, social homage, are awarded the gifted women who excel in song, in the drama, in music, in sculpture, in painting, in literature, in eloquence, in poetry.

A Kellogg, a Neilson, an Urso, a Ream, a Bonheur, a Stowe, a Dickinson, a Browning: these be all bright particular stars, each in her own constellation, sharing alike with her masculine compeers, in the firmament of our age, her full meed of praise. No hero on our battle fields won brighter laurels nor more enduring fame than did the Nightingale of memory yet sweet to countless thousands of the rank and file.

There are Gifts and Gifts. Gifts of the head, the heart, the hand, the arm, aye, even the foot. The thoughtful head—the sympathetic heart—the skillful hand,—the powerful arm—the subtle foot. What taught the old, old fable of the trunk and the members? Certainly, the lesson that not one member, whether of the body physical, or the body social, or the body politic, should be honored for its own excellence alone ; but for the mutual aid that one gives the other, each should be awarded equal honor as the indispensable adjunct of a great and useful economy.

Whatever is useful is worthy—whatever is honorable is worthy—whatever is beautiful is worthy. Shall the honorable or the beautiful say to the useful, "*cui bono?*" God forbid!

He who fashions with his own hands an implement by which the harvester husbands time, saves labor, gets money, is accounted great in a way. He whose skilled hands turn a piece of mechanism delicately wrought to aid the transmission of thoughts and even words to far off lands, is great in another way. He whose brawny arm can lift aloft the ponderous sledge and let it fall adroitly, has drawn encomiums from great brained lookers on, and is great in still another way. Eight thousand pairs of gleaming eyes have betrayed the intense admiration of as many minds in the execution of a difficult *pas*, and an Ellsler leaves the stage amid the wild encore of voice and hands and feet.

Oh! inconsistent Society! Where are your vociferous plaudits, when fingers no less skillful have wrought a marvel of a garment, arrayed and adorned in which you flaunt your presence in the eyes of admiring men and envious women? Why are your choicest terms of praise withheld when she who presides over the secrets of your cuisine, astonishes yourself and guests with a miracle of gustatory excellence? Her's is a questionable fame who wins the custom of the beau monde, and challenges without fear the skill of competitor, in the make up of a head dress. No price in cash is thought exhorbitant, when she demands; but you will not *see* her should you chance to meet her on the walk. By and by grown rich on your vanity, she owns a brown stone front and equipage, and makes no more bonnets. *Then* you exchange calls, and mayhap become proud of you acquaintance with her. How odd!

A girl, braving the edict of Society; yearning after independence; abhorring the position of a cypher when there is a posibility of becoming at least a unit in life; asks a place behind the counter—gets it—likes it—works up into an accountant—is trusted with responsible duties—proves worthy of them—works into a partnership or launches alone upon a mercantile career. She has successfully done what thousands of braggart men have failed to do ; and yet her demand for a full recognition of a position such as we would freely accord a man of like success, is met by an evasion, if not by a point blank refusal.

We honor a *man* if he learns an honest trade and excels therein. Men grown great are proverbially proud of an obscure origin.—Franklin never resented an allusion to his type-setting days, and his ink-fingered period, and his starving times. The Pulpit, the Bar, Physic, Politics, Manufacturers, Science, Art, Literature, are all full of examples of small beginnings and large achievements. Honor, renown, and wealth follow quick upon the heels of success. Johnny and Kitty in the nursery are so taught—John and Catherine hear it in the home circle—have it thundered at them from the rostrum—listen to it in the cushioned pew. It is as preached, a golden rule, with no exceptions—as practiced, its exceptions are legion.

Social geography has a system of maps which is peculiar. Its isothermal lines are exceedingly serpentine, to say the least.—Grand divisions are not wholly clear, even to the closest examiner, and minor details are wretchedly obscure. There are far too many arbitrary lines both of latitude and longitude, and parallels are far from being true to their title, and meridians belie their names.

Let's see : it's the correct thing to be rich ; men must become so by labor of some sort—woman by fate of some sort. It's the correct thing to have business or occupation ; men must let every one know what they do—women must let no one know. It's the correct thing to be a great artist, in song, music, painting, sculpture, the drama ; but it's beneath women to enter upon the steps necessary to reach correct pre-eminence therein. Every stickler for the rights of Society, so called, will throw overboard the girl who asserts her intention of going into any professional study or work ; but so soon as the victim has achieved any success therein, straightway hundreds of high-toned sticklers get upon their aristocratic knees and beg her to shed some of her radiance upon their empty heads. In other words : don't sing until you are a Lind—don't paint until you are a Bonheur—don't model until you are a Ream—don't act until you are a Neilson ; but having gone through the disgrace necessary to become one of these, "come to our dear, useless, aristocratic arms, you sweet pet of Society."

But then you might be the best cook in creation, and never be a heroine—the best seamstress in the land, and reap no word of honorable mention—the best housekeeper in the State and be not much of anybody after all—the sought after by the whole neighborhood for advice in illness and bodily extremity, and nobody for all that—the oracle in fancy and worsted works for all the region round, and thereby be no whit the more acceptable in a social way.

And yet none deny the absolute utility of all these accomplishments in the economy of Society. Think of our dispositions, were the cooks all clumsy ; reflect upon our grotesque presentment were all seamstresses on a long strike; imagine what our home life would be were the art of housekeeping suddenly obliterated from the known world ; depict, if you can, the consternation of having no one to call upon for any help before you can get a doctor out of his bed or away from his dangerous patient ; suppose for an instant that with all your material on hand, there is no one with whom you can confer as to the best and prettiest method of making up your next new prodigy in double zephyr. Alas ! alack !

All this is bosh? *You* don't do these things? Have I not seen it, and thought about it, and mentally condemned it my life long ? I know it is just as I have put it, only a deal worse ; and know, moreover, that it will go on so, ever so long—long as there is a disposition among women to make work and study and responsibility a burden. It *is* easier to be a butterfly than a bee, but the bee has the best of it when the winter comes. Winter to the bee is necessity to the woman. It's all Summer so long as papa furnishes the table with luxuries, the house with elegancies, your person with clothing and books and means of enjoyment. Winter *might* come, though, and you be caught a butterfly.

Statues are pretty to look upon, but I prefer them of stone ; not of flesh and blood.—Automata interest for a period, but become monotonous with familiarity. Wooden ones can be set aside and brought out fresh after a time ; while the doll-daughters won't be put off so. I have often wondered how much credit is due these animated dolls for the sumptuous homes of which they boast.—

Wonder how 'twould be if papa were to become a pauper to-morrow.

Confidential: So long as otherwise sensible men take pretty dolls for better or worse; like a lottery ticket, that *may* turn up a prize, but is pretty sure to be a blank; so long as mamma, knowing just what a risk *she* ran, is willing to let the daughter run the same risk: so long as papa don't put his foot down and say his daughter *must* have an aim in life; so long as no respectable number of women in Society will break the spell that is over them, and determine to be worth something to somebody besides themselves: so long will just such people as the writer continue to bother over the subject, and just such people as his readers smile over the possibility of his thinking he can make the matter better by his screeds. It is some satisfaction, however, to let people know that you know what they know, and that you don't know that they know that you know what they don't know.

